# Differential Diagnosis of Parkinson Disease, Essential Tremor, and Enhanced Physiological Tremor with the Tremor Analysis of EMG

**DOI:** 10.1155/2017/1597907

**Published:** 2017-08-14

**Authors:** Jie Zhang, Yan Xing, Xiuli Ma, Liqun Feng

**Affiliations:** ^1^Aviation General Hospital, China Medical University, Beijing 10023, China; ^2^Anzhen Hospital, Capital Medical University, Beijing 10029, China

## Abstract

We investigate the differential diagnostic value of tremor analysis of EMG on Parkinson's disease (PD), essential tremor (ET), and enhanced physiological tremor (EPT). Clinical data from 25 patients with PD, 20 patients with ET, and 20 patients with EPT were collected. The tremor frequency and muscle contraction pattern of the resting, posture, and 500 g and 1000 g overload were recorded. The frequency of PD tremor was 4–6 Hz, and the frequency of ET was also in this range; the frequency of EPT is 6–12 hz having some overlap with PD. The muscle contraction patterns of the ET and EPT group were mainly synchronous contraction, and the muscle contraction mode of the PD group was mainly alternating contraction. Having tremor latency from rest to postural position and having changes in tremor amplitude after mental concentration in PD might distinguish ET. Tremor analysis of EMG was able to distinguish PD from ET and EPT by varying the tremor frequency and muscle contraction pattern. It can also differentiate between PD and ET by the latency and concentration effect and ET and EPT by weight load effect.

## 1. Introduction

Parkinson's disease tremor is a common type of tremor. In addition to the clinical manifestations, the tremor of Parkinson's disease is lack of relative objective diagnostic basis. The diagnosis of PD and its differentiation from other types of tremor such as ET and EPT are frequently difficult [1]. Especially in early disease stages it remains problematic because tremor in PD may occur not only at rest, but also at posture and/or during action. On the other hand, tremor at rest is not pathognomonic for PD [2], for it has been observed in ET [3]. It is also difficult to differentiate mild ET from EPT. One study suggested that about 50% of clinical diagnoses of ET are incorrect [4]. The early misdiagnosis rate of Parkinson's disease that only displays the tremor was 25% [5]. It is necessary to identify the ET and PD. Because of the different prognosis of the three kinds of diseases, it is urgent to use a kind of examination method to identify the early stage of PD and ET and EPT. The purpose of this study was to investigate the value of the tremor analysis of EMG in the differential diagnosis of PD and ET as well as EPT.

## 2. Clinical Data and Methods

### 2.1. Clinical Data

Clinical data were collected from January 2015 to November 2015 in Aviation General Hospital neurology department with tremor as the main manifestation of Parkinson's disease in 25 patients (PD group). There were 13 males and 12 females, aged 45 to 76 years with an average age of 64.1 years. Duration of PD ranges from 6 months to 4 years with an average of 2.7 years. At the same time, 20 patients with ET were selected (ET group). Tremor is the only clinical manifestation. There were 9 males and 11 females, aged 39 to 82 years. Duration of ET ranges from 2 to 10 years with an average age of 60 years, with an average of 6.2 years. 20 patients were with EPT tremor (EPT group). There were 12 males and 8 females, with the age of 28–74 years and average 51 years. Duration of EPT ranges from 0.3 to 2 years with an average of 0.9 years.

### 2.2. Methods

The Medelec Synergy EMG machine of Oxford company was used in this study with 4 EMG surface electrodes and 1 pressure accelerator [6]. The surface electrodes were applied to the muscles of the flexor carpi ulnaris and the extensor carpi ulnaris, respectively. The electrode was fixed at the distal side of the third metacarpal bone in the same hand. The frequency and amplitude of patients' tremor were recorded when they were in the sitting position at rest (put relaxed hand on thighs), in posture position (double forearm straight forward), and in forearm loaded by 500 g and 1000 g, respectively. Each recording last for 30 s. Patients were observed if they had tremor immediately from static to postural changes to determine whether tremor had latency and were asked to continuously calculate the subtraction “100-7” when postural tremor occurred in order to determine whether mental activity had an effect on tremor.

#### 2.2.1. Data Analysis

Internationally recognized tremor analysis software (TRAS system) was used for the analysis of the EMG activity data and tremor frequency. The frequency range of tremor was between 1 and 30 Hz. Tremulous energy is recorded as amplitude and simultaneous recording of EMG activity associated with tremor.

#### 2.2.2. Statistic Treatment

SPSS 16 software was used to analyze the data. The data were expressed as mean ± standard deviation (*χ* ± s), and the *t*-test was used. *P* < 0.05 was statistically significant.

## 3. Result

**Figure 1:**
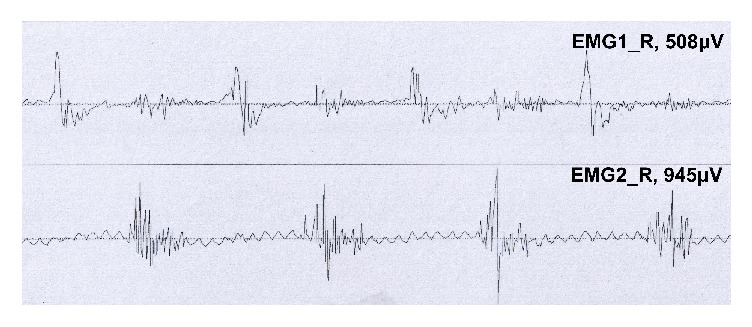
Patterns of muscle contraction in Parkinson's disease tremor.

**Figure 2:**
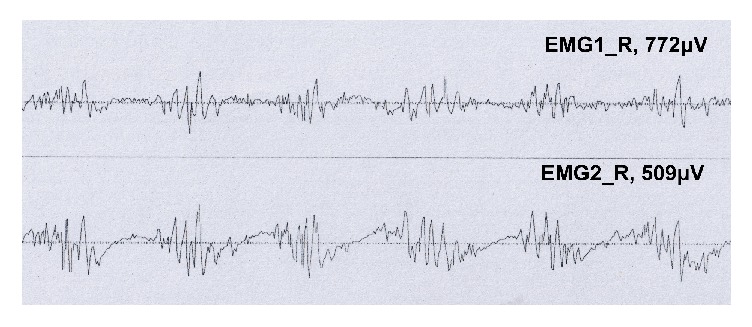
Patterns of muscle contraction in essential tremor.

In 20 patients with ET, there were 6 patients with static tremor by naked eye. In 25 patients with PD, there were 9 patients with visible postural tremor; there was 1 patient with visible static tremor in EPT. However, there was a significant increase in ET, PD, and EPT by tremor analysis of EMG, shown in 10 cases, 15 cases, 5 cases, respectively. In PD group, the tremor frequency at rest, posture, and weights 500 g and 1000 g was at about 4–6 Hz. The tremor frequency of ET patients at rest, posture, and weight was about 5–8 Hz. The frequency of EPT was increased to 6–12 Hz. The frequency of EPT was significantly reduced by weight load effect, and the reduction was about 50%. There was no significant difference between the tremor frequency at rest and after loading between PD group and ET group (*P* > 0.05). The tremor frequency of PD group was significantly different from that of EPT for several conditions, including rest, posture, and weight bearing 1000 g (*P* < 0.05 or *P* < 0.01) (Table 1). These results implied that it was difficult to distinguish between PD and ET only through the tremor frequency at rest condition. The main difference between the two groups was that the main tremor muscle contraction pattern of PD was as the main alternative (Figure 1), but ET was for synchronization (Figure 2) and the different changes about concentration effect and latency. Compared with the ET and EPT groups, postural tremor in PD patients was significantly delayed, and mental activity significantly increased the amplitude of PD tremor.

## 4. Discussion

As a common clinical manifestation, tremor can be divided into static tremor, postural tremor, intention tremor, and so on. The common types of tremor mainly include Parkinson's disease tremor and essential tremor. Traditionally, PD is equal to resting tremor, and ET is postural tremor, which is quite one-sided [7]. Parkinson's disease tremor is relatively poor because of its relatively poor prognosis. It is easy to be confused because of some similarities to ET and EPT.

Although patients with Parkinson's disease display tremor, rigidity, bradykinesia, postural abnormalities, and other typical clinical manifestations, still many patients were with atypical clinical manifestations or showed only a symptom. The diagnosis of PD and its differentiation from other types of tremor such as ET and EPT are frequently difficult. Especially in early disease stages it remains problematic. Postural tremor may be the isolated or predominant symptom in early stages of PD, in which rest tremor, bradykinesia, rigidity, and postural stability may be slight or inexistent [8].

Now, the differential diagnoses of PD, ET, and EPT are still mostly based on clinical phenomena. The accurate diagnosis of tremor is of fundamental importance to both patients and clinicians because assessment of prognosis and treatment selection depend on tremor type. It is crucial to the success of therapeutic trials [8].

We found that PD and ET were difficult to distinguish in tremor frequency, and there was a superposition of the tremor frequency, the frequency of PD tremor was 4–6 Hz, and the frequency of ET was also in this range; the frequency of EPT is 6–12 hz having some overlap with PD. We found that although the frequency of PD tremor and ET tremor is difficult to identify, ET has more postural tremor. If static tremor is present, static tremor frequency was generally less than the frequency of postural tremor 1.5–2 hz [8]. Our study found that the difference between the two was 1.8 Hz, also in line with foreign literature reports. And we found no delay of postural tremor from resting to lifting upper limb in ET, and PD had obvious delay. For PD patients, the two muscles of the forearm mainly displayed alternating contraction but synchronous contractions were mostly observed in ET patients. However, ET muscle contraction was mainly synchronous contraction, and alternating contraction was rare. Most of the PD and ET could be identified by the combination of different patterns of muscle contraction. But some scholars believe that the pattern of muscle contraction can show different burst at different times by long-term recording [9]. We found that if there was postural tremor in PD patients, most would have increased tremor amplitude during mental activity. The reason is unknown, but whether this phenomenon is principle similar to Jendrassik manoeuvre in the tendon reflex needs further investigation.

The origin of the tremor of PD is located in the globus pallidus; some scholars believe that the subthalamic nucleus hyperexcitability leads to increased number of cells in the posterior part of the globus pallidus [10]. ET is probably caused by abnormal reticular formation or abnormal nuclear motion in the central nervous system. At present, the pathogenesis of the two diseases is different to extent. A recent comprehensive review suggests that the relative risk of patient with ET to develop PD is 2–4 times higher than in the general population [11]. Of course, in addition to electrophysiology, we also need to ask about family history and so on. One approach may be to use the term essential tremor only as a transitional node in the deep phenotyping of tremor disorders based on historical, phenomenological, and neurophysiological features to facilitate its etiologic diagnosis or serve for future gene and biomarker-discovery efforts [12].

But both PD and ET can show static and postural tremor; only one of them shows one main manifestation, and the frequency of tremor of the two diseases is similar. The tremor analysis of EMG finding more static or postural tremor than by naked eyes may indicate that these two diseases are probably homologous; just resting tremor was activated in PD but was suppressed in ET.

In the early stages of PD, in this study, we find that tremor frequency is stable in patients with central oscillator, such as PD and ET, but unstable in EPT. We can differentiate PD and ET by the latency and concentration effect and ET and EPT by weight load effect. Neurophysiologic tests may help in cases of coexistence of postural and rest tremor. We think some neurophysiologic criteria for the diagnosis of PD are useful: (1) there is rhythmic burst of rest tremor on EMG, (2) tremor frequency is stable at 4–6 Hz; (3) if postural tremor exists, there is tremor latency from rest to postural position; (4) changes of the dominant frequency peak are less than or equal to 1 hz after the weight load test; (5) there are changes in tremor amplitude after mental concentration. EMG tremor analysis can be used to identify Parkinson's disease and essential tremor and enhanced physiological tremor. The method is based on accelerometer and surface electromyography (EMG) electrodes. It is readily available, noninvasive, and cost-efficient diagnostic tool. But due to the small sample statistics, further large sample is needed for further research, and future research work will be further observed after treatment of PD and idiopathic tremor, strengthen the change frequency and patterns of muscle physiological tremor, and be combined with clinical assessment scale.

## 5. Conclusion

Tremor analysis of EMG is suitable for clinical use. It was able to distinguish PD from ET and EPT by varying the tremor frequency and muscle contraction pattern. It can also differentiate PD and ET by the latency and concentration effect and ET and EPT by weight load effect. As a noninvasive, sensitive, and repeated examination technique, it is believed that tremor analysis of EMG should have wider application prospects and research prospects in clinical practice.

## Figures and Tables

**Table 1:** Tremor frequency, latency, concentration effect, and synchronous EMG of Parkinson's disease, essential tremor, and enhanced physiological tremor.

	Resting (Hz)	Posture (Hz)	Loaded 500 g (Hz)	Loaded 1000 g (Hz)	Latency	Concentration effect	Synchronous EMG
ET (*n* = 20)	5.38 ± 1.23	7.32 ± 2.63	6.46 ± 2.04	6.31 ± 1.36	0%	0%	80%
PD (*n* = 25)	4.52 ± 0.43	4.94 ± 0.66	5.17 ± 0.62	4.71 ± 0.96	80%	100%	20%
EPT (*n* = 20)	8.48 ± 4.41	12.6 ± 4.52	7.47 ± 2.81	9.3 ± 1.26	0%	0%	85%

At rest, PD versus EPT *P* < 0.05; postural ET and PD versus EPT *P* < 0.01; ET and PD versus EPT *P* < 0.05 when loading 1000 g; PD versus ET; EPT *P* < 0.05 with mental activity and latency; PD versus EPT *P* < 0.05 with synchronous EMG.
